# RETRACTION: Antioxidant Properties and Cardioprotective Mechanism of Malaysian Propolis in Rats

**DOI:** 10.1155/ecam/9862607

**Published:** 2026-07-28

**Authors:** Evidence-Based Complementary and Alternative Medicine

RETRACTION: R. Ahmed, E. M. Tanvir, M. S. Hossen, et al., “Antioxidant Properties and Cardioprotective Mechanism of Malaysian Propolis in Rats,” *Evidence-Based Complementary and Alternative Medicine*, 2017, 5370545, https://doi.org/10.1155/2017/5370545.

The above article, published online on 02 February 2017 in Wiley Online Library (https://onlinelibrary.wiley.com), has been retracted by John Wiley & Sons Ltd. The retraction has been agreed due to multiple concerns identified in Figure 7, some of which are raised on PubPeer [1]. Further investigation identified additional concerns with the Figure, specifically:

• Panel D appears identical to panel C in Figure 5 of [2].
• Panel C appears identical to panel D in Figure 5 of [2] and to panel C in Figure 3 of [3].

Following an assessment of the concerns raised, together with the author’s response and the absence of original image data, the results and conclusions can no longer be considered reliable. The article is therefore retracted.

The authors agree with the retraction.
